# Knowledge, attitudes and practices (KAP) relating to brucellosis in smallholder dairy farmers in two provinces in Pakistan

**DOI:** 10.1371/journal.pone.0173365

**Published:** 2017-03-16

**Authors:** Shumaila Arif, Peter C. Thomson, Marta Hernandez-Jover, David M. McGill, Hassan Mahmood Warriach, Jane Heller

**Affiliations:** 1 School of Animal and Veterinary Sciences, Charles Sturt University, Wagga Wagga, New South Wales, Australia; 2 Graham Centre for Agricultural Innovation, Charles Sturt University, Wagga Wagga, New South Wales, Australia; 3 School of Life and Environmental Sciences, The University of Sydney, Camden, New South Wales, Australia; 4 ASLP Dairy Project, University of Veterinary & Animal Sciences, Lahore, Pakistan; 5 Faculty of Veterinary and Agricultural Sciences, The University of Melbourne, Melbourne, Victoria, Australia; University Hospital Jena, GERMANY

## Abstract

The present study aimed to assess the extent of knowledge and understanding of brucellosis in smallholder dairy farmers and identify practices at the farm and household level that might pose a risk for humans contracting brucellosis. Between February and June 2015 a cross-sectional study was conducted among smallholder farms (*n* = 420) in five districts of Punjab and two districts of Sindh province. Farmers were interviewed using a questionnaire to obtain information on farmers’ knowledge about brucellosis and the potential risks for contracting the disease that are present for dairy farmers and their families. Logistic regression and ordinal logistic models were used to investigate potential predictors for risky behaviours. The results show almost all farmers (97%) were not aware of the modes of transmission of brucellosis. Relating to risk, the majority (66%) of the farmers’ families were reported to consume raw milk and its products, live in shared housing with animals (49%) and not cover hand cuts during contact with animals (74%). All farmers performed at least one risky practice on a regular basis for brucellosis transmission from animal to human. A multivariable analysis highlighted that the respondents with no formal education and those who had not heard of the disease displayed greater risky behaviour. Poor understanding of the disease, presence of multiple risky practices on farm and at the household, and incorrect perception supports the need for an educational awareness program in order to ensure uptake of improved practices.

## Introduction

Brucellosis is the earliest identified bacterial disease with more than 500,000 human cases every year globally [1]. Historically, it has been called by many names, comprising Malta fever, Mediterranean fever, undulant fever and crops disease in humans or Bang’s disease in cattle [2]. The Food and Agriculture Organization (FAO), World Health Organization (WHO) and World Organization for Animal Health (OIE) consider brucellosis as one of the most pervasive zoonoses in the world [3].

Brucellosis is caused by different species of the genus *Brucella*. The major species of *Brucella* and their hosts are: *B*. *abortus* (cattle), *B*. *melitensis* (goats), *B*. *suis* (pigs) and *B*. *ovis* (sheep). Human brucellosis can be caused by *B*, *abortus*, *B*. *meliensis* and *B*. *suis* [4]. These pathogens are intracellular and persist within an individual animal, resulting in life time carriage of the organism [5]. In livestock, brucellosis mainly affects sexually mature animals and causes late trimester abortions, weak calves and infertility characterized by placentitis and epididimitis. Diseased animals shed the pathogen in uterine discharge, vaginal discharge and milk [6] and these bacteria can spread within the herd through ingestion of contaminated material [7].

Brucellosis can be considered to be a disease of animals, where humans are accidental hosts. The disease in humans results from ingestion or inhalation of the pathogen, or direct entrance via skin abrasions [8]. Humans can get the disease through consumption of raw milk and raw milk products from infected animals and via direct handling with contaminated materials from infected animals, specifically in aborted foetuses, foetal membranes and vaginal secretions. As a result, people who have frequent contact with animals in areas where brucellosis is endemic are at high risk of contracting the disease [9]. Symptoms of brucellosis in human are not specific, but most patients with the acute form of the disease report fever, sweats, malaise, anorexia, headache, arthralgia, arthritis, and backache [10]. It can develop serious complication of epididymoorchitis among affected male patients [11].

Although bovine brucellosis has been eradicated in many high-income countries in Europe and in Australia, Canada, Israel, Japan and New Zealand [12], it is still not controlled in areas such as Africa, the Middle East and Asia [13], where the disease is endemic. In many, low-income countries the prevalence of human and animal brucellosis is increasing and the dearth of awareness, policies and resources are the main contributor to this development [1]. The World Animal Health Information Database [14] maintained by the OIE, states there are many clinical cases in the Middle East and Africa and Latin America but no data are available about Pakistan.

In low-income countries like Pakistan, dairy animals are important for the livelihood of rural communities [15]. Human brucellosis is a neglected and under-recognized disease in these countries [16]. In Pakistan, only a few studies have been conducted to estimate the prevalence of brucellosis in humans, ranging from 6.9% to 21.7% among veterinary professionals and abattoir workers, respectively [17, 18]. Similarly, limited studies have been carried out regarding bovine brucellosis and have reported inconsistent prevalence. A recent sero-prevalence study from Pakistan reported 6–15% prevalence of bovine brucellosis under three different management systems [19]. However, the available prevalence estimates are on large farms and these estimates are not generalizable for the predominant system in Pakistan, which is a smallholder structure. So there is still need to study the disease burden in smallholder farming system. The majority (90%) of the dairy industry is based on smallholder farms [20] which is associated with a number of people that have their health and economics affected if the disease is present in the system.

The current study aimed to assess the extent of knowledge and understanding of brucellosis in smallholder dairy farmers in two provinces in Pakistan and identify practices at the farm and household level that might pose a risk for humans contracting brucellosis. It is expected that the findings of this study will helpful to devise future disease control programs and one health interventions.

## Material & methods

This study was conducted in collaboration with the ASLP (Agriculture Sector Linkages Program) dairy extension research project (LPS/2010/2007) through the Australian Centre for International Agricultural Research (ACIAR). The ASLP project aims to strengthen the dairy value chains in Pakistan through improved farm management and more effective extension services. The ASLP project has been implemented in seven districts in Pakistan, and involves 8 to 10 villages in each district, with a group of 15 to 20 farmers in each village. The ASLP dairy project farmers are getting benefit in terms of education and training at regular intervals on whole-farming systems (basic husbandry, nutrition, reproduction and calf rearing) to improve the farm production through farmer discussion groups [21].

### Study area, design and selection of participants

**Fig 1:**
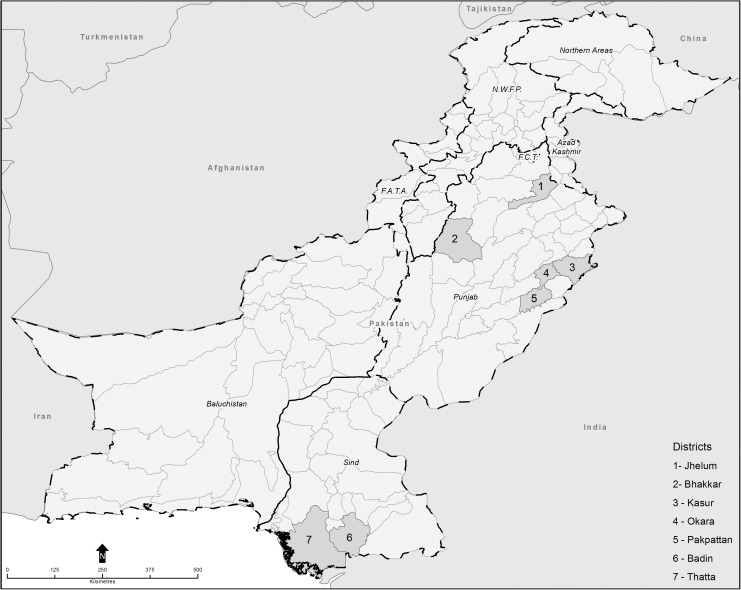
Map of Pakistan indicating current study areas (Map was created using ArcGIS® software by Esri. ArcGIS® and ArcMap™)

Between February and June 2015, a cross-sectional study using an interview-based survey was conducted among smallholder dairy farmers. This cross-sectional study was carried out in five districts of Punjab (Okara, Pakpattan, Kasur, Jhelum, and Bhakkar) and two districts of Sindh (Thatta and Badin) (Fig 1).

These districts were selected on the basis of operational convenience, but also represented a range of social and physical differences across the two provinces of Pakistan. The demographic characteristics of these districts are described in Table 1. These areas are dominated by smallholder dairy farmers, who own less than 10 cattle and buffaloes [20].

**Table 1:** Demographic characteristics of the study districts of Pakistan. Source: Pakistan Bureau of Statistics (2015).

Province	District	Area	Population			
		(km^2^)				
			Number	Density	Rural	Urban
				(per km^2^)	%	%
**Punjab**	Kasur	3,995	2,375,875	594.9	77.1	22.8
	Okara	4,377	2,232,992	510.2	76.9	23.0
	Pakpattan	2,724	1,286,680	472.3	85.7	14.2
	Jhelum	3,587	936,957	261.2	72.3	27.6
	Bhakkar	8,153	1,051,456	107.5	83.9	16.0
	Overall	22,836	7,883,960	345.2	78.8	21.1
**Sindh**	Thatta	6,726	1,136,044	168.9	83.5	16.4
	Badin	17,355	1,113,194	64.1	88.7	11.2
	Overall	24,081	2,249,238	93.4	86.1	13.8

Information regarding the name of the village, number of farmers in the village and village leader contact details was received from the ASLP dairy project field staff and local veterinarians. In the first step, two villages from each district were selected randomly using a random number generation method in Microsoft Office Excel (Microsoft, PC/windows XP, 2010, Redmond WA, USA). Two villages in Sindh were replaced with nearby villages as animals were sent for grazing and the village leaders were reluctant to participate in the study. Note that two villages per district were sampled to obtain what could be considered a representative sample across the district. While sampling more villages might be desirable, logistics of the study limited expansion to more villages. In the second step, dairy farms and households were selected (as described below) and a farm and household was considered one study unit. The sample size calculation was based on an unknown prevalence (thus assumed to be 50%) of brucellosis among study units. This study was conducted concurrently with another study estimating the seroprevalence of bovine brucellosis in Pakistan. The sample size was calculated with Epitools (http://epitools.ausvet.com.au/content.php?page=PrevalenceSS). Accordingly, a total of 431 study units were identified to be required for this study. Selection of the households was done with the help of focal farmers (village leader / farmer representative) in each village. Note that while a random selection of farmers from a village might be desirable, it was necessary to work with the focal farmers in the selection of households to ensure cooperation of farmers. A total of 431 farmers were targeted for interviews, with approximately 30 farmers per village being selected.

A structured questionnaire (available on request from the corresponding author) comprising closed (*n* = 56) and open-ended (*n* = 8) questions, was used to gather information on farmers, knowledge about brucellosis which is known as “Athra” in Urdu and Punjabi languages and “Tunbjan” in Sindhi language, and potential risks of contracting brucellosis among dairy farmers and their families. For closed questions, participants were asked to indicate from a pre-existing set of answers.

The questionnaire comprised two parts. The first part included questions about demographic characteristics, knowledge about animal brucellosis clinical signs of brucellosis in cattle and buffalo, and potential herd management practices that could pose a risk for brucellosis acquisition in animals. The second part of the questionnaire focused on knowledge of human brucellosis, potential routes of transmission from animals to people and information on practices posing a risk of brucellosis acquisition in humans within the household. Major risky practices associated with herd management and in the household included in the questionnaire are listed in Table 2. The questionnaire was pretested to assess clarity and time requirement by 15 farmers and modified in line with feedback from the pre-test. Questions regarding clinical signs of human brucellosis were not included due to being a sensitive topic culturally and the nonspecific and diverse nature of clinical presentations. The final version of the questionnaire was uploaded on iSurvey^TM^ (https://www.harvestyourdata.com/), a survey tool that allows data to be collected offline on portable tablet devices.

**Table 2:** Farm and household practices which pose risk of contracting brucellosis in animals and humans, included in a questionnaire among smallholder dairy farmers in Pakistan.

Herd management practices	Household practices
Dung cleaning*	Consume raw milk and its products
Feeding and water trough cleaning*	Live in shared place animals
Store dung piles for more than 6 months	Cover hand cuts while contact with placental membrane*
Wash udder before milking*	Direct contact with placental/aborted foetus material while handling parturition
Disinfect space after parturition*	Wash hands before and after milking*
Dispose of placental membranes after parturition/aborted foetus material*	
Shared calving space with other animals	
Slaughter animals at farm	
Send animal for common grazing	

*The absence of this practice is considered risky

### Interview procedure

Each village was visited for 3–4 days and 10–13 farmers were interviewed each day. On the first day of arrival in each village, the study team organised a farmer discussion group (FDG), with separate groups for male and female farmers. In each FDG, the study team explained the objectives and the participant information sheet in their local languages (Punjabi, Sindhi, and Urdu). Farmers were told that participation in the study was absolutely voluntary, and that the identification of the farm/herd/household would not be disclosed. At the end of FDG meeting, farmers were requested to nominate the family members working the most with animals, for participating in the interview. If a female farmer was responsible for the most farm and household work then she was interviewed for the full questionnaire. However, if the male was responsible for most of the work in the farm and the female (wife, sister or mother) was in the house then the questionnaire was completed by both, each completing the questions in relation to their main responsibilities.

### Ethics statement

The focal farmers contacted two to three times via mobile phone to explain the study aims and request for collaboration in the current study. They were then requested to contact farmers and identify suitable days for implementation of the interviews. Farmer’s discussion groups were organised with focal farmer consent. All the participants were informed about the aims of the study, methods, and the individual information will be not be disclosed, and voluntary participation. The participant information sheet was explained to the farmer groups prior to starting individual interviews. Written consent with the participant’s signature was not possible because many of the farmers were not able to read the consent form. Verbal consent was recorded on the consent form after participant agreement. The current study, participant information sheet, and the consent form/method were approved by the Charles Sturt University Human Research Ethics Committee (Australia; Approval Number 2014/222).

### Statistical analysis

Factors associated with risky herd management practices were investigated by means of initial descriptive statistics as well as Venn diagrams to show patterns of multiple risky practices. This was followed by logistic regression modelling to assess factors associated with the practices. The herd management practices (Table 1) were investigated. The response variables were coded as 1 (Yes) vs 0 (No). Explanatory variables tested for association were 1) District, 2) Age, 3) Education, 4) Knowledge of whether farmers can get diseases from animals (No / Not sure / Yes); 5) Heard about brucellosis in humans (No / Not sure / Yes); 6) Knowledge of brucellosis transmission (No / Yes); and 7) Knowledge that raw milk is a source of brucellosis (No / Yes). Initial univariable logistic regression models were fitted for all response variable and explanatory variable combinations. Explanatory variables with *P* < 0.20 in the univariable analyses were considered further in the multivariable analyses. A backwards elimination procedure was used to build the final multivariable models for each of the outcome variables. Explanatory variables with *P* < 0.10 were included in the final model, although associations with 0.05 < *P* < 0.10 were considered as suggestive only. Note that District was not included in the final model, due to potential confounding with other explanatory variables, and hence inability to assess their biological significance. Results from the final models were expressed in terms of odds ratios with associated 95% confidence intervals. In a similar way, factors associated with household practices explained in Table 2 were also investigated. Model fitting was conducted using the glm() function in R (R Core Team, 2015).

In addition, three risk practice scores were calculated, being the total number of risky practices undertaken by a farmer in three categories:

1. Farm cleaning risk score: Farm practices, which farmers routinely do to clean the farm. The farm cleaning risk score (scored from 0 to 4) was the total number of risky practices done by the farmer within the following four herd management practices (Table 2) not cleaning up dung; 2) not cleaning the feeding trough; 3) storage of dung piles for more than 6 months; and, 4) not washing udder before milking.
2. Brucellosis herd transmission risk score: This metric identified potential factors posing a risk for brucellosis acquisition in animals. The brucellosis risk score (scored from 0 to 5) was based on the number of the following risky herd management practices: 1) common grazing for animals; 2) not disinfecting space after birth; 3) not disposing of placental membranes; 4) calving space shared with other animals; and, 5) slaughter of animals on-farm. Unlike the farm cleaning risk score, the brucellosis herd transmission risk score includes what can be considered high-risk factors particularly associated with brucellosis transmission within herd.
3. Household risk score: Household risky practices include unhygienic practices, which pose risk of *Brucella* transmission to humans. Household risk practice score (scored from 0 to 5) was the total number of risky household practices among those mentioned in Table 2

Note that the herd management practices listed in Table 2 have been divided into two sets for these risk scores. These three sets of scores were treated as ordinal scale variables, and hence, factors associated with these scores were analysed using ordinal logistic regression. For all scores, the same sets of explanatory variables considered in the individual risk practices analyses listed above were used.

In addition, to assess the overall association between the household risk score (response variable) and the two herd management risk scores (i.e. farm cleaning risk score and brucellosis herd transmission risk score, both categorical explanatory variables), an additional ordinal logistic regression model was fitted to the data. The function clm()in the ordinal package of R was used for these ordinal models [22].

## Results

Of the 431 selected smallholder dairy farmers, seven were not available for the interview and four refused to participate in the study. As a consequence a total of 420 dairy farmers participated in the study.

### Demographic characteristics of the respondents

The majority (64%) of farms were run by female members of the households. Overall, 46% of respondents had no formal education, while 12% reached intermediate (completed high school) or above. More than half of the households were comprised of 6–10 family members with 7.5% of households having over 15 members. Most of the participants were in the 25–54 age groups (Table 3).

**Table 3:** Demographic features of smallholder dairy farmers of Pakistan participating in a cross-sectional study on brucellosis (*n* = 420 farmers).

	Category	Percentage
Performs most of the work on-farm	Male	36
	Female	64
Level of education	No formal education	46
	Primary	14
	Middle	12
	Matric	16
	Intermediate	6.8
	University	5.2
Number per household	1–5	18
	6–10	57
	11–15	17
	>15	7.5
Age	10–24	11
	25–34	18
	35–44	30
	45–54	22
	55+	18

### Knowledge of the respondents regarding brucellosis

The majority of participants had heard of animal brucellosis (70%), with only a quarter of participants having heard about human brucellosis (26%). Although 23% of farmers recognised that they can get any disease from animals, only 3% of farmers were aware of the modes (raw milk, contact with aborted foetus or placental membrane) of brucellosis transmission from animal to human (Table 4).

**Table 4:** Knowledge and understanding about brucellosis among smallholder dairy farmers of Pakistan participating in a cross-sectional study on brucellosis (*n* = 420 farmers)

	Category	Percentage
Heard about the brucellosis as an animal disease	Yes	70
	No	30
Knows that farmers can get any diseases from animals	Yes	23
	No	60
	Not sure	17
Heard about the brucellosis as human disease	Yes	26
	No	21
Knowledge about the modes (raw milk, contact with aborted foetus or placental membrane) of brucellosis transmission from animals to human	Yes	3
	No	97
Knowledge about raw milk as a source of brucellosis transmission	Yes	16
	No	62
	Not sure	22

### Herd management practices

**Fig 2:**
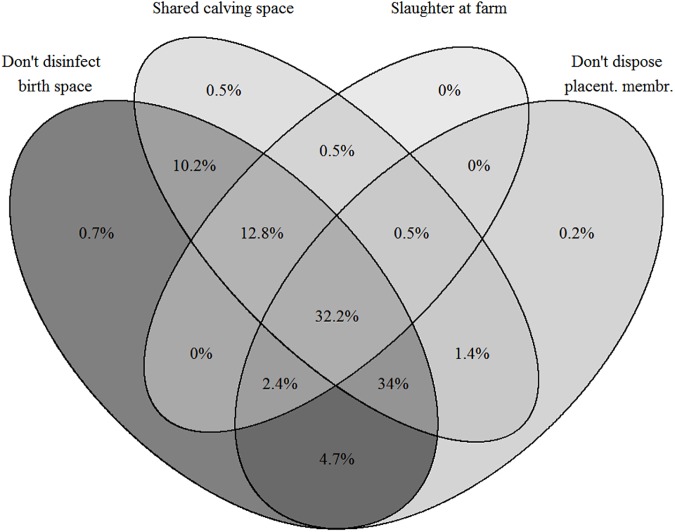
Venn diagram showing the percentages of smallholder dairy farmers in Pakistan having combinations of multiple herd management practices posing a risk of brucellosis transmission within herd.

Most participants (92%) reported that they do not have separate space or shed for parturition and calving, and the space was shared with other animals. The majority (86%) of farmers stored dung piles for more than six months at the farm. Only 24% of farmers properly disposed placental membranes by burying them (Table 5). All farmers reported at least one risky herd management practice and 32% of participants were undertaking multiple practices which has shown as overlap in Fig 2, namely sharing calving space, not disposing properly of the placental membranes, slaughtering animals on the farm, and not disinfecting the space after birth.

**Table 5:** Herd management practices posing a risk for brucellosis transmission within herd reported by smallholder dairy farmers participating in a cross-section study on brucellosis in Pakistan (*n* = 420 farmers).

Herd Management Practices	Percentage
Dung cleaning*	64
Feeding and water trough cleaning*	76
Store dung piles for more than 6 mounts	86
Animal access to dung piles	70
Send animal for common grazing	56
Disinfectant space after parturition*	2.3
Dispose placenta membranes and aborted foetus by burring*	24
Shared calving space with other animals	92
Slaughter animals at farm	48

*The absence of this practice is considered risky

The univariable logistic regression models showed that all herd management practices with the exception of “Disinfect space after birth” had significant differences in occurrence across the districts (*P* < 0.001). Table 6 shows that Thatta district had higher risk for several herd management practices, whereas Bhakkar had lower risk for most of the practices. Further, Okara had increased risk of dung pile storage for more than six months compared to other districts. Note that the two Sindh districts (Badin, Thatta) had much lower levels of prevalence of slaughtering animals at farm, compared with the five Punjab districts (Kasur, Okara, Pakpattan, Jhelum, and Bhakkar). Results for the final multivariable models are shown in Table 7; note that the outcome variables shown are only for those which had at least one significant predictor variable (*P* < 0.05) in the final model, and District was not included in the models, as noted previously. In general respondents with no formal education showed high risk levels, being less likely to dispose placental membranes (*P* = 0.0001) and Slaughtering animals at farm dispose of placental membranes (*P =* 0.002) appropriately.

**Table 6:** Summary of the univariable models for herd management risky practices showing the effect of District. Columns of the table refer to specific practices (outcome variables) and rows refer to particular districts. Shown are the odds ratio (OR) and 95% confidence interval (95% CI) for the OR, relative to the reference district Badin in the Sindh province (OR = 1).

**District**	**Dung cleaning**		**Feeding and water trough cleaning**		**Dung pile storage**		**Wash udder**		**Disinfect space after birth**	
	***P* = 2.08e-07**		***P* = 9.90e-06**		***P* = 5.36e-06**		***P* = 1.33e-07**		***P* = 0.47**	
	**OR**	**95% CI**	**OR**	**95% CI**	**OR**	**95% CI**	**OR**	**95% CI**	**OR**	**95% CI**
Badin	1		1		1		1		1	
Bhakkar	1.31	(0.56, 3.03)	2.4	(0.98, 5.92)	1.18	(0.37, 3.76)	1.00	(0.06, 16.34)	1.52	(0.24, 9.46)
Jhelum	0.92	(0.41, 2.04)	1.90	(0.81, 4.46)	1.02	(0.32, 3.04)	0.49	(0.04, 5.56)	9.38	(0 Inf)
Kasur	0.33	(0.15,0.71)	1.67	(0.72,3.87)	0.22	(0.02, 0.57)	0.31	(0.03, 3.13)	1.55	(0.25, 9.63)
Okara	0.54	(0.24,1.17)	2.61	(1.03,6.61)	7.73	(0.88, 62.41)	0.04	(0.005, 0.34)	1.05	(0.14, 7.73)
Pakpattan	1.61	(0.67,3.84)	2.41	(0.98,5.92)	0.58	(0.21, 1.63)	0.06	(0.0-+08, 0.54)	4.91	(0.04,5.56)
Thatta	0.23	(0.10, 0.49)	0.40	(0.19,0.85)	1.41	(0.43, 4.85)	0.04	(0.04,5.56)	1.00	(0.13, 7.33)
**District**	**Dispose placental membrane**		**Shared calving space**		**Common grazing area**		**Slaughtering animals at farm**			
	***P* = 2.2e-16**		***P* = 0.002**		***P* = < 2.2e-16**		***P* < 2.2e-16**			
	**OR**	**95% CI**	**OR**	**95% CI**	**OR**	**95% CI**	**OR**	**95% CI**		
Badin	1		1		1		1			
Bhakkar	45.47	(10.14, 203)	1.03	(0, Inf)	0.01	(0.00, 0.06)	17.26	(6.04, 49.30)		
Jhelum	16.64	(3.70, 74.80)	1.72	(0.39, 7.56)	1.08	(0.49, 2.33)	13.20	(4.64, 37.50)		
Kasur	17.07	(3.79, 76.83)	8.03	(0.23, 2.78)	0.16	(0.07, 0.36)	28.32	(9.68, 82.87)		
Okara	8.52	(1.82, 39.68)	4.28	(0.13, 1.34)	2.17	(0.90, 5.18)	11.20	(3.92, 31.99)		
Pakpattan	1.0	(0.13, 7.33)	2.63	(0.49, 14.13)	0.46	(0.22, 0.97)	26.75	(9.19, 77.81)		
Thatta	3.21	(0.62, 16.62)	5.91	(0.18, 1.92)	5.06	(1.74, 14.31)	3.33	(1.11, 9.94)		

**Table 7:** Summary of the final multivariable models for herd management risky practices. Columns of the table refer to specific practices (outcome variables) and rows refer to predictor variables after the backward elimination process. *P*-values are shown for each predictor variable, followed by the odds ratio (OR) and 95% confidence interval (95% CI) for the OR, relative to the reference group (OR = 1). No entries (——) indicate where there was no significant association in the final multivariable model.

	Dung cleaning		Feeding and water troughs cleaning		Dispose placental membrane		Shared calving space		Slaughtering animals at farm		Common grazing area	
	OR	95% CI	OR	95% CI	OR	95% CI	OR	95% CI	OR	95% CI	OR	95% CI
**Education**	——		——		***P* = 0.0001**		——		***P* = 0.002**		——	
- No formal					1				1			
- Primary					1.28	(0.60, 2.72)			1.26	(068, 2.33)		
- Middle					3.65	(1.84, 7.26)			1.44	(0.75,2.77)		
- Matric					3.41	(1.81, 6.41)			1.23	(0.69,2.20)		
- Intermediate					0.99	(0.34, 2.86)			0.41	(0.17,0.98)		
- Bachelor					2.65	(0.98, 7.17)			0.20	(0.06, 0.64)		
**Think they can get disease**	——		***P* = 6.7e-05**		——				***P* = 0.001**		——	
- No			1						1			
- Not Sure			2.87	(1.34, 6.12)					1.89	(1.09,3.29)		
- Yes			3.46	(1.69, 7.08)					2.29	(1.39, 3.76)		
**Heard about animal brucellosis**	——	——	***P* = 0.0039**			——	***P* = 0.0002**		***P* = 0.02**		***P* = 0.01**	
No				1			1		1		1	
Yes			2.05	(1.26, 3.33)			3.86	(1.88,7.92)	1.68	(1.06, 2.66)	0.52	(0.33,0.81)
**Heard about human brucellosis**	——	——	——		***P* = 0.06**		***P* = 0.020**		——		——	
- No					1		1					
- Not Sure					0.71	(0.07,6.64)	1.21	(0.21, 6.96)				
- Yes					1.82	(1.09,3.04)	3.89	(2.16, 7.00)				
**Knowledge about disease transmission**	***P* = 0.07**		***P* = 0.02**		***P* = 0.07**		——		——		——	
- No	1		1		1							
- Yes	3.33	(0.73, 15.08)	2.83	(0, Inf)	2.79	(0.90, 8.66)						

Respondents who knew that they could get diseases from livestock were more likely to clean the feeding trough (OR = 3.46 for ‘feeding and water troughs’ and *P* = 6.78e-05), but were also more likely to slaughter animals at the farm (OR = 2.29 and *P* = 0.0012). Further, the participants who had heard about animal brucellosis were more likely to clean feeding troughs (OR = 3.46 and *P* = 0.004), but they were also more likely to practice ‘shared calving space’, ‘slaughter animal at farm’ and send their animals for common grazing. Farmers who heard about human brucellosis were marginally more likely to dispose placental membranes. Finally, and somewhat expectedly, respondents who understood how brucellosis was transmitted showed reduced levels of several risky herd practices (Table 7): they were marginally more likely to clean the dung pile (OR = 3.33 for ‘dung cleaning’ and *P* = 0.076), more likely to clean troughs (OR = 2.83 and for feeding and water troughs cleaning and *P* = 0.02), and marginally more likely to dispose of the placental membranes.

### Household practices

**Fig 3:**
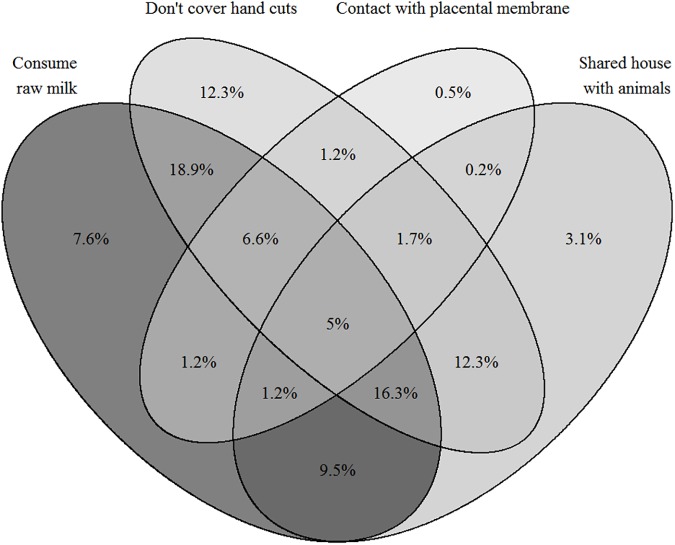
Venn diagram showing the percentages of smallholder dairy farmers in Pakistan having combinations of multiple household practices posing a risk of brucellosis transmission from animals to human.

The majority (66%) of the farmers’ families consume raw milk and its products, almost half of the participants live in shared housing with animals and do not cover hand cuts during contact with animals (74%) (Table 8). Overall, 16.3% of all respondents reported three household practices which pose risk of brucellosis acquisition and all farmers reported at least one risky practice (Fig 3).

**Table 8:** Household practices that pose risk for brucellosis transmission reported by smallholder dairy farmers participating in a cross-section study on brucellosis in Pakistan (*n* = 420 farmers).

Household Practices	Percentage
Consume raw milk and its products	66
Live in shared place with animals	49
Cover hand cuts while contact with animals	25
Direct contact with placental membrane while handling parturition	17
Wash hands before and after milking*	61

*The absence of this practice is considered risky

The univariable logistic regression models showed that for all household practices with the exception of “Direct contact with placental membrane”, there were significant differences across the districts (all *P* < 0.001, Table 9). However, no clear overall trend of districts is evident, with specific risks being evident in specific districts (e.g. living in shared places with animals in Thatta; not covering hand cuts in Pakpattan).

**Table 9:** Summary of the univariable models for household practices showing the effect of District. Columns of the table refer to specific practices (outcome variables) and rows refer to particular districts. Shown are the odds ratio (OR) and 95% confidence interval (95% CI) for the OR, relative to the reference district Badin in the Sindh province (OR = 1).

District	Use raw milk and its products		Live in shared place with animals		Cover hand cuts while contact with animals		Direct contact with placental membrane		Wash hands before and after milking	
	*P* = 1.87e-06		*P* < 2.2e-16		*P* = 0.0009		*P* = 0.24		*P* < 2.2e-16	
	OR	95% CI	OR	95% CI	OR	95% CI	OR	95% CI	OR	95% CI
Badin	1		1		1		1		1	
Bhakkar	0.27	(0.12, 0.57)	0.39	(0.18, 0.81)	0.42	(0.19, 0.94)	0.98	(0.34, 2.86)	3.25	(1.35, 7.85)
Jhelum	2.61	(1.07, 6.37)	0.07	(0.02, 0.18)	0.39	(0.17, 0.86)	1.97	(0.76, 5.11)	1.15	(0.54, 2.44)
Kasur	0.63	(0.30, 1.33)	0.68	(0.33, 1.42)	0.52	(0.24, 1.127)	0.73	(0.23, 2.26)	6.20	(2.16, 17.08)
Okara	1.42	(0.63, 3.19)	0.21	(0.09, 0.46)	0.37	(0.16, 0.84)	1.72	(0.64, 4.59)	0.10	(0.04, 0.25)
Pakpattan	0.92	(0.43, 1.98)	0.81	(0.39 1.68)	0.15	(0.05, 0.42)	1.45	(0.54, 3.91)	0.23	(0.11, 0.50)
Thatta	1.08	(0.49, 2.33)	3.25	(1.35, 7.85)	0.87	(0.42, 1.80)	2.16	(0.84, 5.55)	2.08	(0.93, 4.66)

As shown in Table 10, the multivariable model shows that respondents with no formal education were more likely to live in shared places with animals compared to the farmers with Middle- and Matric-level education (*P* < 0.0001). Further, they were less likely to cover their hand cuts while in contact with animals (*P* = 0.007). Those respondents who had heard about human brucellosis were more likely to cover their hand cuts while with animals (OR = 2.05 and *P* = 0.016) and they were marginally less likely to have direct contact with placental membranes (OR = 0.62 and *P* = 0.052). Farmers who were aware of raw milk being a source of disease tended not to consume raw milk (OR = 0.48 and *P* = 0.006) but to boil it before consumption. Also of note is an increase in the reporting of having direct contact with the placenta with increasing respondent age (OR = 4.30 and *P* = 0.007).

**Table 10:** Summary of the final multivariable models for household risky practices. Columns of the table refer to specific practices (outcome variables) and rows refer to predictor variables after the backward elimination process. *P*-values are shown for each predictor variable, followed by the odds ratio (OR) and 95% confidence interval (95% CI) for the OR, relative to the reference group (OR = 1). No entries (——) indicate where there was no significant association in the final multivariable model.

	Use raw milk and its products		Live in shared place with animals		Cover hand cuts while contact with animals		Direct contact with placental membrane		Wash hands before and after milking	
	OR	95% CI	OR	95% CI	OR	95% CI	OR	95% CI	OR	95% CI
**Education**	——		***P* = 1.7e-07**		***P* = 0.007**		——		——	
- No formal			1		1					
- Primary			1.04	(0.56, 1.94)	2.44	(1.22,4.87)				
- Middle			0.28	(0.14, 0.55)	1.86	(0.90, 3.86)				
- Matric			0.22	(0.11, 0.41)	2.13	(1.11, 4.06)				
- Intermediate			0.29	(0.12, 0.69)	3.89	(1.62, 9.34)				
- Bachelor			0.53	(0.21, 1.32)	0.82	(0.22, 3.02)				
**Age**	——			——		——	***P* = 0.04**		——	
- <25							1			
- 25–34							1.52	(0.49, 4.65)		
- 35–44							1.12	(0.38, 3.29)		
- 45–54							2.21	(0.77, 6.37)		
- 55+							3.19	(1.41, 9.18)		
**Think they can get disease from animal**		——	***P* = 0.00015**		***P* < 0.0001**			——		——
- No			1		1					
- Not Sure			0.32	(0.17, 0.58)	0.09	(0.03,0.29)				
- Yes			0.52	(0.31, 0.87)	0.75	(0.43, 1.29)				
**Heard about human brucellosis**		——				***P* = 0.016**	***P* = 0.052**		——	
- No					1		1			
- Not Sure					2.66	(0.41, 17.19)	3.6	(0.71, 18.26)		
- Yes					2.05	(1.23, 3.41)	0.62	(0.34, 1.15)		
**Know raw milk is source of disease**	***P* = 0.0060**			——		——		——	***P* = 0.005**	
- No	1								1	
- Yes	0.47	(0.28, 0.80)							2.24	(1.23, 4.08)

### Farm cleaning risk score

Univariable analyses identified that the farm cleaning risk score (i.e. total number of risky practices in that category) is significantly associated with District, and ‘Knowledge of whether farmers can get diseases from animals’ (both *P* < 0.001), as well as with Education level, and ‘Heard about animal brucellosis’ (both *P* < 0.05). Considering the difference between districts, the highest (worst) farm cleaning scores were obtained in Okara (Punjab) and Thatta (Sindh), whereas, the remaining five districts presented low risk levels (Figure A in S1 File). Furthermore, the final multivariable model shows that the “Education” and ‘‘Knowledge of whether farmers can get diseases from animals” were significant explanatory variables (Table 11). The farmers with an average level (Matric) of education were having lower scores than the highest education level. In addition, the participants with the knowledge that they can get disease from animals were undertaking better cleaning practices (Table 11, Figure B in S1 File).

**Table 11:** Summary of the final multivariable models for the three risk scores. Columns of the table refer to specific risk scores (outcome variables) and rows refer to predictor variables after the backward elimination process. *P*-values are shown for each predictor variable, followed by the odds ratio (OR) and 95% confidence interval (95% CI) for the OR, relative to the reference group (OR = 1). OR from the ordinal logistic model refer to the odds of obtaining a certain score of higher, compared to a lower score, so OR > 1 implies increased risk.

	Farm cleaning risk score		Brucellosis herd transmission risk score		Household risk score	
	OR	95% CI	OR	95% CI	OR	95% CI
	*P* = 0.016		——		*P* = 3.2e-06	
**Education**						
- No formal	1				1	1
- Primary	0.58	(0.33, 1.02)			0.56	(0.33, 0.96)
- Middle	0.57	(0.32, 1.01)			0.36	(0.20, 0.64)
- Matric	0.41	(0.24, 0.71)			0.24	(0.14, 0.42)
- Intermediate	0.50	(0.23, 1.07)			0.41	(0.20, 0.83)
- Bachelor	0.61	(0.26, 1.47)			0.49	(0.21, 1.12)
**Think farmer gets diseases from animals**	***P* = 0.00011**		——		***P* = 0.048**	
- No	1				1	
- Not Sure	0.52	(0.31, 0.88)			1.67	(1.02, 2.72)
- Yes	0.40	(0.25, 0.63)			0.84	(0.54, 1.30)
**Heard about brucellosis in humans**	——		***P* = 0.0061**		——	
- No			1			
Not sure			0.35	(0.07,1.61)		
-Yes			0.54	(0.36,0.81)		
**Age**	——		——		***P* = 0.042**	
- 10–24					1	
- 25–34					1.61	(0.84, 3.10)
- 35–44					0.72	(0.39, 1.34)
- 45–54					1.03	(0.53, 2.00)
- 55+					1.18	(0.60, 2.34)

### Brucellosis herd transmission risk score

Univariable ordinal analyses showed highly significant associations between the brucellosis herd transmission risk score with District and ‘Heard about human brucellosis’ (both *P <* 0.01). Comparing districts, Pakpattan and Okara had the highest (i.e. worst) scores while Bhakkar had the lowest scores (Figure C in S1 File). In the multivariable analysis, only the explanatory variable ‘Heard about human brucellosis’ was significant, with farmers who had not heard about brucellosis in humans having higher risk scores (Table 11).

### Household risk score

Univariable ordinal analyses showed highly significant associations between the household risk score with District, Education and Age (all *P* < 0.01). Comparing districts, similar to the brucellosis herd transmission risk score for animal brucellosis, Pakpattan and Okara had the highest scores, while Bhakkar had the lowest scores (Figure D in S1 File).When considering the multivariable model, again Education, Age but also ‘Knowledge of whether farmers can get diseases from animals’ were significantly associated with the household risk scores (*P* = 0.048). There were lower (i.e. better) scores with increasing education levels up to ‘Matric’, but increasing scores at the highest education levels (Figure E in S1 File), as was also observed in the farm cleaning risk scores. Further, farmers that were not sure about potential transmission of disease from animals had higher household risk scores than farmers that were aware and middle age participants had better household practices when compared to other age groups (Table 11).

### Association between the household and herd management risk scores

**Fig 4:**
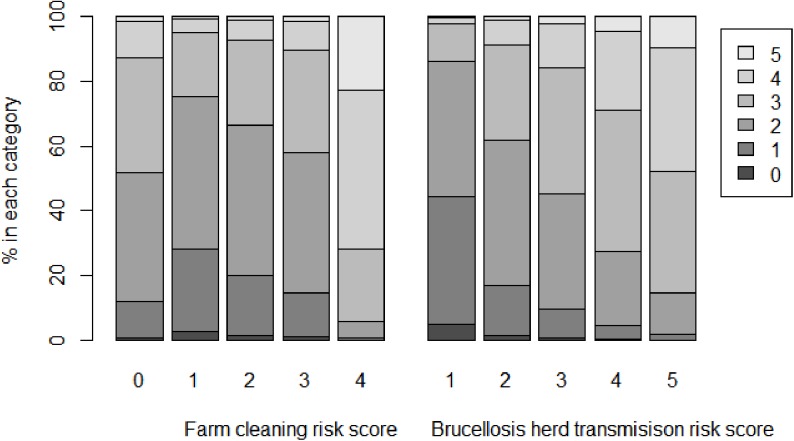
Model-based percentages in each household risk score category (0, 1, 2, 3, 4, 5) across levels of farm cleaning risk scores, and across levels of brucellosis herd transmission risk scores. These values are from the two-variable model and are averaged over the levels of the other factor in the model. Household risk scores and brucellosis herd transmission risk scores were calculated from the practices reported by smallholder dairy farmers who participated in a cross-sectional study on brucellosis in Pakistan

The association between the two herd management risk scores (Farm cleaning risk score and Brucellosis herd transmission risk score) and the household risk score was investigated and the univariable models and two-variable model indicate that both were significantly associated with the outcome (both *P* < 0.001, Fig 4). The respondents who were doing better farm cleaning and brucellosis risk-mitigation practices at farm level were more likely to do better practices at household level as well.

### Self-reported practices and unsubstantiated beliefs

Twenty four percent of the participants reported consuming raw milk and its products in summer and 11% of them sell raw milk to their neighbours on a regular basis. Close to one third of the participants reported throwing aborted foetuses and placental membranes in dung piles and a few reported throwing aborted foetuses in a water canal or river. Thirteen percent of respondents believed that abortion in cattle and buffalo was associated with having a woman who had aborted living in proximity of the farm. Sixteen percent of participants reported seeking help from shrines for the treatment of brucellosis in animals (Table 12).

**Table 12:** Descriptive results of self-reported practices and unsubstantiated beliefs among smallholder farmers of Punjab and Sindh, Pakistan.

Self-reported practices and unsubstantiated beliefs	Percentage
Consume raw milk and its products in summer only	24
Sell raw milk to neighbours	11
Throw aborted foetus and placental membrane in dung piles	34
Throw aborted foetus in canal water	4
Send their animals for common grazing in summer only	15
Believe any type of abortion in cattle and buffalo as bovine brucellosis	57
Believe last trimester abortion in cattle and buffalo is caused by if an aborted women is in proximity of the herd	13
Seek brucellosis treatment from shrines	16

## Discussion

The current study revealed that the knowledge and understanding about brucellosis among smallholder farmers in Pakistan is limited. To our knowledge, this is the first study conducted on smallholder farmers’ knowledge and awareness about brucellosis in Pakistan. The majority of participants had heard about animal brucellosis (70%) but few had heard about human brucellosis (26%), which agrees with the study results reported in Uganda [23], Egypt [24], and Jordan [25]. In contrast, studies from Tajikistan [26] and Kenya [27] showed low awareness about the disease among farmers in these areas. The low awareness of human brucellosis in the present study could be attributed to the dearth of health education (especially regarding zoonotic diseases) and the low proportion of farmers receiving formal education in the study regions. Further, in the current study, only approximately a quarter of respondents were aware of the risk of transmission of any disease from animals to humans, however the respondents with knowledge about disease transmission were more likely to perform better preventive practices to avoid contracting brucellosis. These results are supported by previous literature. Kozukeev [28] in a study in Kyrgyzstan explained that good knowledge about the disease transmission routes for brucellosis among farmers had a precautionary effect for brucellosis. In a similar way, a case control study in Iran demonstrated that having awareness regarding modes of brucellosis transmission, i.e. consumption of raw milk cheese, was associated with a reduced risk of human brucellosis infection [29]. This suggests that improving farmers’ knowledge of the disease and its mode of transmission is likely to reduce their risk of brucellosis transmission from animals.

Findings from current study illustrate that despite the majority of respondents having heard about animal brucellosis, all respondents were engaged in at least one practice at their farm and household conducive to transmission of *Brucella* to other animals and most importantly to humans. Knowledge about the disease and preventive herd management practices have previously been identified as the most important factors required for minimising the disease risk in animals [30]. Most farmers reported calving space being shared with other animals, and a study by [31] shows the importance of this practice for brucellosis transmission to other animals. Many *Brucella* organisms are shed during the 10 days after calving or at abortion, contaminating the environment, and increasing the risk of other cattle and buffalo ingesting the organism [32]. Only one-third of farmers in this study reported disposing placental membranes by burying, with most disposing them in dung piles or feeding them to other animals. In addition, the majority of farmers reported storing dung piles for more than six months and allowing their animals to access to these dung piles. Given that *Brucella* can survive in a humid environment (manure and soil) for several months, this may also represent a risk for disease transmission in animals [19]. More than half of the participants reported sending their animals to common grazing places with other village animals, which could also represent a risk for herd-to-herd transmission. Community pastures have been previously described as a risk for disease transmission and the management of such pastures should be considered when implementing any control measures [33].

In relation to practices posing a risk for brucellosis transmission from animals to humans, consumption of raw milk has been previously described as one the most risky practices [34]. In the current study, two-thirds of respondents reported that they consumed raw milk (unpasteurized) and its products on a regular basis, suggesting a higher risk transmission of brucellosis, if the disease is present in the herd. For some of the respondents, the reason for drinking raw milk in hot summer periods was the belief that raw milk has a cooling effect. Another important risky practice for contracting brucellosis is humans sharing accommodation with animals [9]. Almost half of the respondents in the present study lived in shared accommodation with animals.

This study confirms that the level of formal education is associated with the knowledge of brucellosis and the practices in relation to this disease. Participants with no formal education were less likely to be aware of brucellosis in comparison to those with middle or matric-level of education. However, participants with intermediate- or university-level education reported riskier herd management practices, perhaps because the farming aspects were not as central to their livelihoods, thus being of lower priority in their everyday activities. Farmers with no or lesser level of education were less likely to have good hygienic practices at their homes and were putting themselves at more risk of contracting brucellosis. This is also illustrated by the studies conducted in Yemen [35] and in Tajikistan [26]. Furthermore, in the present study, analysis of risky practice scores demonstrates that respondents with formal education had positive influences on farm cleaning and household practices, as opposed to respondents with no formal education. However, this difference between formal and no formal education was lower than expected. This could be explained by the lack of health education on zoonotic diseases in the formal education system. Similarly, respondents with knowledge about brucellosis had a lower risk practice score. Analysis of risky practices scores indicates that knowledge of the disease is very important and it is the overall predictor of farmer’s behaviour towards risk. Good knowledge of the disease is associated with lower risk scores, indicative of practices that would not support disease transmission. This study also found that the level of risk differs across the seven districts. However, none of the districts were risk-free and if the disease is present in the region, it would spread quickly within communities due to the prevalence of risky practices both at farm and household levels. Findings from the district-wise analysis indicate the need for targeted educational interventions according to risk level of the district. Further, the results obtained here can be used to customise particular educational programs for different districts, according to their risk profiles.

It is important to note that the majority of participants are not aware of the risk of zoonotic diseases being transmitted from domestic animals to humans, especially from cattle and buffalo. Although awareness of brucellosis in animals was high, farmers did not think they could get the same disease from their animals. Farmer perceptions about brucellosis are guided by cultural and religious beliefs, and may often be ill-informed and inappropriate. Many participants discussed different unsubstantiated beliefs (i.e. ‘myths’) about the cause of brucellosis in animals and the majority of participants would go to *Darbar* (shrines) for seeking help with their animals’ abortions or bring Taweez (Holy verses) for treatment. These cultural beliefs would need to be considered in relation to any educational intervention program.

Findings from this study demonstrate a poor understanding of brucellosis and a high level of risky practices being undertaken on farm and at the household across a number of regions, all contributing to the risk of humans contracting brucellosis. Poor knowledge of the disease and of practices posing a risk of disease transmission, incorrect perceptions and attitudes towards the management of the disease, such as the use of shrines, strongly supports the need for health education in rural communities. For low-income countries like Pakistan, targeted and culturally appropriate health education is required to increase the awareness of not only brucellosis but other zoonotic diseases. Education is the feasible preventive measure, where testing and slaughtering of infected cattle and buffalo is not an economically or socially viable option and milk pasteurization is not common practice at smallholder level. A synergistic “one health” approach to this type of education in rural communities would be ideal in order to ensure uptake of recommendations and practice change on a farm and household level.

## Supporting information

S1 File A-D Figs.
(PDF)
