# Supplementary material for: Knowledge, attitudes and practices (KAP) relating to brucellosis in smallholder dairy farmers in two provinces in Pakistan
Source: PLoS One. 2017 Mar 16;12(3):e0173365. doi: 10.1371/journal.pone.0173365 (PMC5354373; doi:10.1371/journal.pone.0173365)
Supplement: S1 File — A-D Figs. (PDF) [file pone.0173365.s001.pdf]

Supporting Information

Knowledge, attitudes and practices (KAP) relating to brucellosis in smallholder farmers in two provinces in Pakistan

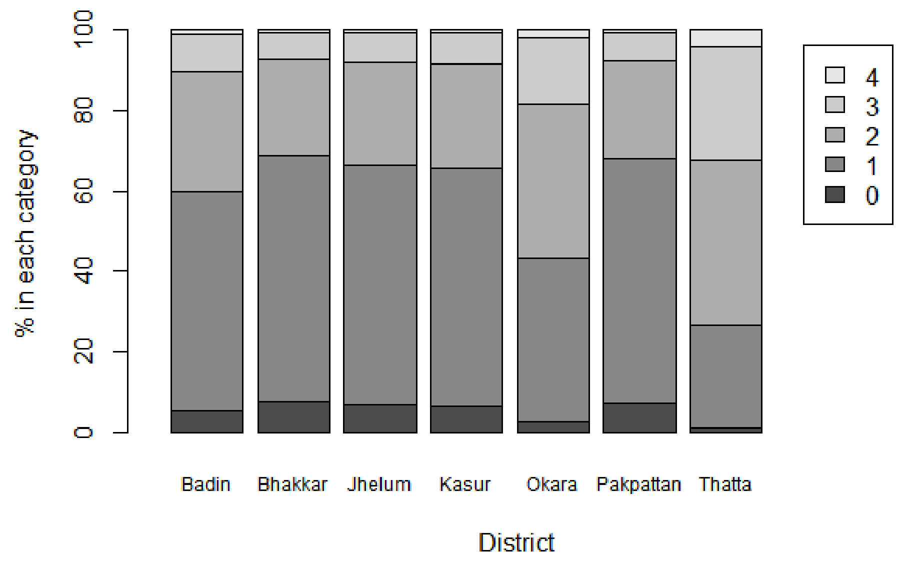

SI-Figure A. Bar plot showing model-based percentages in each farm cleaning risk score category (0, 1, 2, 3, 4) across the seven districts. These values are from the univariable model. The farm cleaning risk score was calculated from the herd management practices reported by smallholder dairy farmers who participated in a cross-sectional study on brucellosis in Pakistan.

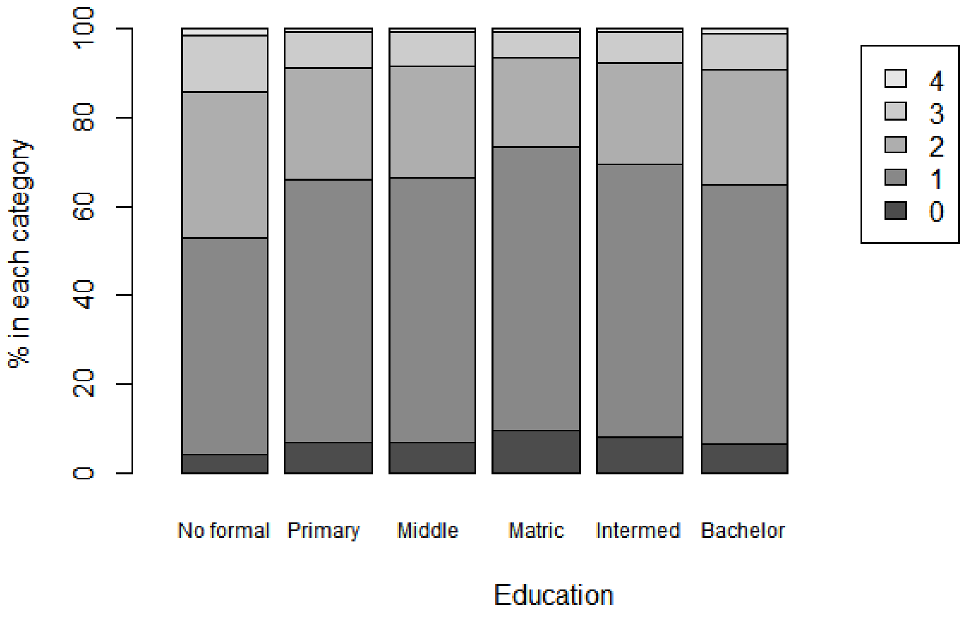

SI- Figure B. Bar plot showing model-based percentages in each farm cleaning risk score category (0, 1, 2, 3, and 4) across the levels of farmer's education. These values are from the final multivariable model and are averaged over the levels of the other factor in the model. The farm cleaning risk score was calculated from the herd management practices reported by smallholder dairy farmers who participated in a cross-sectional study on brucellosis in Pakistan.

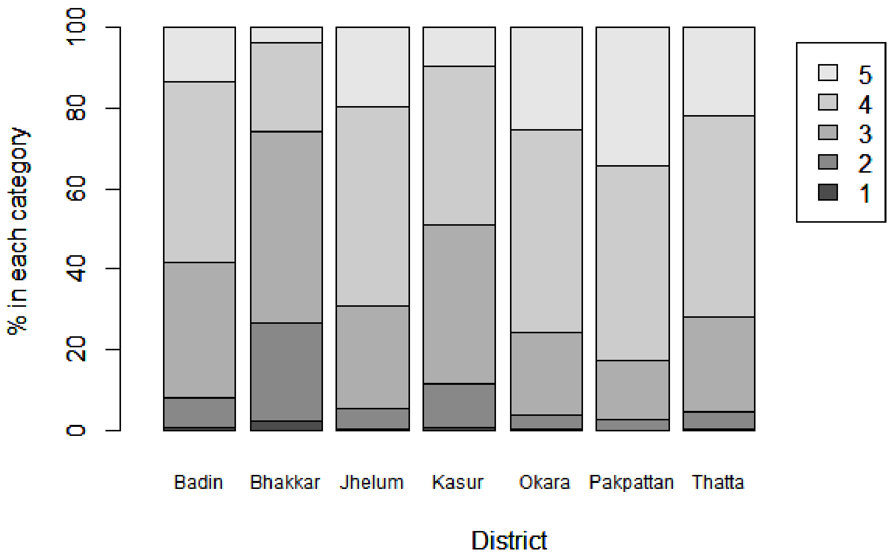

SI-Figure C. Bar plot showing model-based percentages in each brucellosis herd transmission risk score for category (1, 2, 3, 4, and 5) across the seven districts. No scores of zero were recorded. These values are from the univariable model. The brucellosis herd transmission risk score was calculated from the herd management practices reported by smallholder dairy farmers who participated in a cross-sectional study on brucellosis in Pakistan.

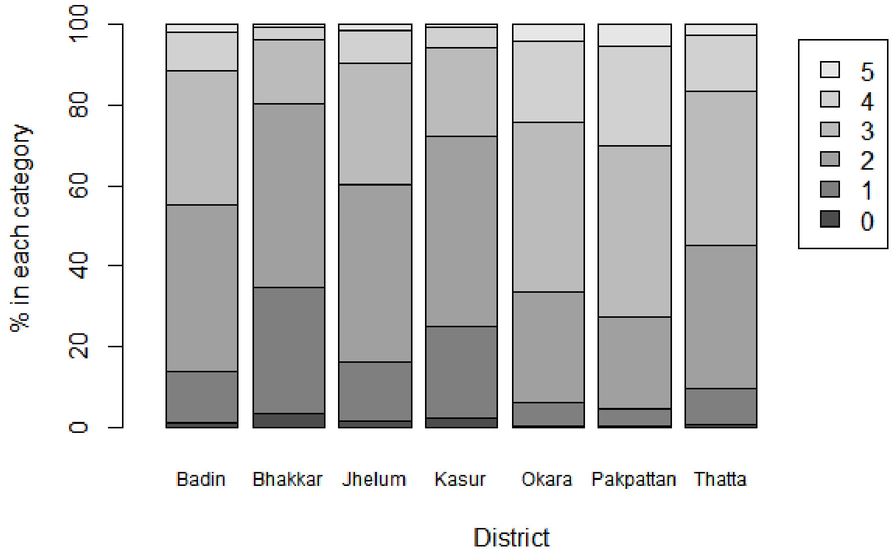

SI-Figure D. Bar plot showing model-based percentages in each household risk score category (0, 1, 2, 3, 4, and 5) across the seven districts. These values are from the univariable model. The brucellosis herd transmission risk score was calculated from the household practices reported by smallholder dairy farmers who participated in a cross-sectional study on brucellosis in Pakistan.

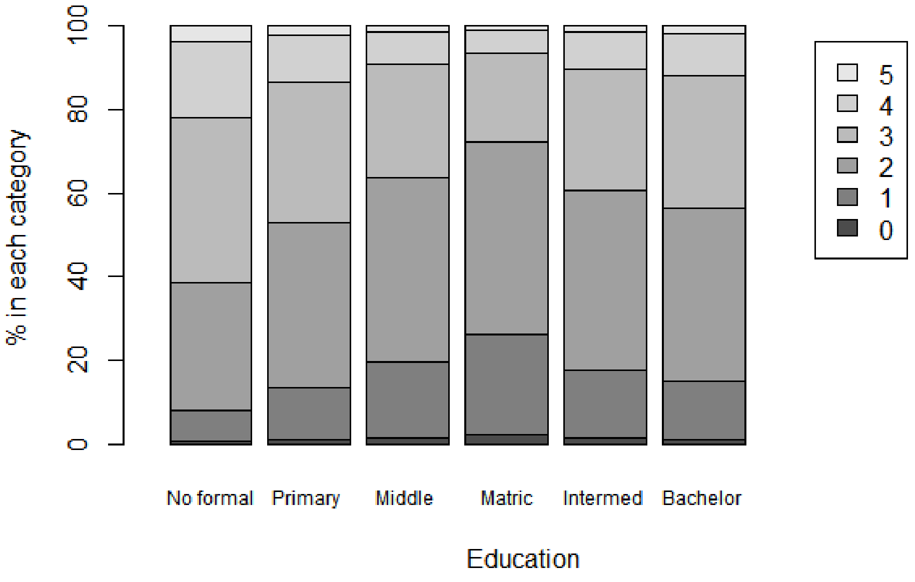

SI-Figure E. Bar plot showing model-based percentages in each household risk score category (0, 1, 2, 3, 4, and 5) across the levels of farmer's education. These values are from the final multivariable model and are averaged over the levels of the other factors in the model. The household risk score was calculated from the household practices reported by smallholder dairy farmers who participated in a cross-sectional study on brucellosis in Pakistan.
